# Ocean acidification and temperature increase impact mussel shell shape and thickness: problematic for protection?

**DOI:** 10.1002/ece3.1756

**Published:** 2015-10-12

**Authors:** Susan C. Fitzer, Liberty Vittert, Adrian Bowman, Nicholas A. Kamenos, Vernon R. Phoenix, Maggie Cusack

**Affiliations:** ^1^ School of Geographical and Earth SciencesUniversity of GlasgowGlasgowG12 8QQUK; ^2^ School of Mathematics and StatisticsUniversity of GlasgowGlasgowG12 8QQUK

**Keywords:** Biomineralization, CO_2_, mussels, ocean acidification, shell shape, shell thickness, temperature

## Abstract

Ocean acidification threatens organisms that produce calcium carbonate shells by potentially generating an under‐saturated carbonate environment. Resultant reduced calcification and growth, and subsequent dissolution of exoskeletons, would raise concerns over the ability of the shell to provide protection for the marine organism under ocean acidification and increased temperatures. We examined the impact of combined ocean acidification and temperature increase on shell formation of the economically important edible mussel *Mytilus edulis*. Shell growth and thickness along with a shell thickness index and shape analysis were determined. The ability of *M. edulis* to produce a functional protective shell after 9 months of experimental culture under ocean acidification and increasing temperatures (380, 550, 750, 1000 *μ*atm *p*CO_2_, and 750, 1000 *μ*atm *p*CO_2_ + 2°C) was assessed. Mussel shells grown under ocean acidification conditions displayed significant reductions in shell aragonite thickness, shell thickness index, and changes to shell shape (750, 1000 *μ*atm *p*CO_2_) compared to those shells grown under ambient conditions (380 *μ*atm *p*CO_2_). Ocean acidification resulted in rounder, flatter mussel shells with thinner aragonite layers likely to be more vulnerable to fracture under changing environments and predation. The changes in shape presented here could present a compensatory mechanism to enhance protection against predators and changing environments under ocean acidification when mussels are unable to grow thicker shells. Here, we present the first assessment of mussel shell shape to determine implications for functional protection under ocean acidification.

## Introduction

Organisms that form calcium carbonate shells are considered most at risk from ocean acidification (IPPC, 2007; Doney et al. 2009): the under‐saturation of carbonate resulting in reduced calcification and growth (Beniash et al. 1997; Doney et al. 2009; Thomsen and Melzner 2010; Thomsen et al. 2013) and potential for subsequent shell dissolution (Kamenos et al. 2013). Reduced growth in *Mytilus edulis* grown in the Baltic sea under natural conditions resembling future ocean acidification (Thomsen and Melzner 2010; Thomsen et al. 2013) occurred under much more variable coastal conditions (Thomsen et al. 2013). The growth of *M. edulis* was also reduced under ocean acidification laboratory experiments based on environmental conditions of an estuarine coastal Loch Fyne (Fitzer et al. 2015), similar to the Baltic Sea. The Baltic Sea and Loch Fyne have similar variability, as freshwater inputs into the catchments reduce the total alkalinity, pH, and carbonate concentrations, providing conditions consistent with early‐onset ocean acidification (Jansson et al. 2013). It has been reported that ocean acidification will impact not only growth but also the ultrastructure of mollusk shells (Dickinson et al. 2012; Ivanina et al. 2013; Coleman et al. 2014; Fitzer et al. 2014b), echinoderms (Byrne et al. 2014), and coralline algal skeletons (Kamenos et al. 2013). A reduction in the force required to crush the sea urchin, *Tripneustes gratilla*, was noted when exposed to ocean acidification and warming conditions, raising concerns for the skeleton protective function (Byrne et al. 2014). Mechanical integrity of shells has been reported to decline with ocean acidification with reduced microhardness after 15 weeks (Ivanina et al. 2013) and fracture resistance after 11 weeks (Dickinson et al. 2012) in the juvenile oyster *Crassostrea virginica* (Dickinson et al. 2012; Ivanina et al. 2013) and reduced fracture toughness in adult mussel *M. edulis* (Fitzer et al. 2015). The impact of ocean acidification on the organism is likely to be as a result of reduced organism control over biomineralization, which was observed in the mussel *M. edulis* (Fitzer et al. 2014b). Altered structural integrity of mussel shells could impact the ability of organisms to survive under changing environments and predation (Fitzer et al. 2015). This raises questions about the ability of the shell to provide protection for the marine organism under ocean acidification and increasing temperatures.

The common blue edible mussel *M. edulis* is an economically important species and an important foundation species for the ecosystem ideal for investigation of the ability of calcifying organisms to produce a protective shell during changing environments. The *M. edulis* bivalved shell closes to protect the organism against predation and desiccation under changing intertidal estuarine environments. Phenotypic plasticity of shell shape and morphology has been used previously to compare functional morphology between Mytilids, *Crenomytilus grayanus*,* Mytilus coruscus*, and *Modiolus modiolus*, in varying spatial coastal distributions relating to changing environmental factors (Vekhova 2013). The shell shape and sculpture of freshwater mussels have been explained by hydrologic variability (Hornbach et al. 2010). In the quagga mussel, *Dreissena bugensis*, phenotypic plasticity in shell morphology occurred in response to increases (18–20°C) or decreases (6–8°C) in temperature (Peyer et al. 2010). Morphological shell plasticity in the freshwater snail *Radix balthica* was induced by the presence of predators, resulting in a more rotund shell with a low spire for increased survival against shell crushing predators (Brönmark et al. 2011). It appears that shell shape plasticity can change with environmental conditions and may be a good indicator of environmental change related to shell function (Hornbach et al. 2010; Peyer et al. 2010; Brönmark et al. 2011; Vekhova 2013). Ocean acidification reduces the ability of *M. edulis* to produce proteins for biomineralization, impacting shell growth (Fitzer et al. 2014b). Under ocean acidification, changes to growth could impact the protective function of the shell. Morphological changes such as increasing shell thickness and production of a more rotund shell shape have been used by organisms as a defensive mechanism to combat predators (Brönmark et al. 2011; Naddafi and Rudstam 2014). Here, we investigate how long‐term (9 months) ocean acidification (550, 750, 1000 *μ*atm *p*CO_2_) and increasing temperatures (750 and 1000 *μ*atm *p*CO_2_ + 2°C) impact the shape of the *M. edulis* shell in comparison with the mussel shell growth and shell thickness.

## Materials and Methods

### Mussel collection and culture

Mussels (*M. edulis*) were obtained from Loch Fyne (Loch Fyne Oyster rope culture), Argyll, UK (Loch Fyne Oysters Ltd), during October 2012. Mussels (1 year old) were cultured in experimental tanks (6 L) supplied with natural filtered (1 *μ*m and UV) seawater at seasonally changing Loch Fyne temperatures and ambient *p*CO_2_ (~380 *μ*atm *p*CO_2_) or ocean acidification (550, 750, 1000 *μ*atm *p*CO_2_) and increased temperature (750 and 1000 *μ*atm *p*CO_2_ + 2°C) conditions for 9 months. The mussels included in this study to examine the impact of warming at 9 months were for 750 and 1000 *μ*atm *p*CO_2_ + 2°C, and not for the control (380 *μ*atm *p*CO_2_) plus warming, due to reduced mussels available for analyses. Four separate 6‐L tanks were fed from one sump system with two sump “header” tanks supplying 8 tanks per treatment. Mussel shells were stained prior to experimental culture using seawater containing the fluorescent dye calcein (150 mg L^−1^ calcein C0875‐25 g; Sigma‐Aldrich^®^, Sigma‐Aldrich Company Ltd., Dorset, England, www.sigmaaldrich.com.) (Fitzer et al. 2014b). Mussels were fed 10 mL of cultured microalgae (five species of algae, *Nannochloropsis sp*., *Tetraselmis sp*., *Isochrysis sp*., *Pavlova sp*., *Thalassiosira weissflogii* (stock from Reefphtyo, UK)) per tank every other day (Fitzer et al. 2014b). The feeding regime (10 mL of ~2.8 million cells mL^−1^ algae culture) was equivalent to ~4666 cells mL^−1^ during experimental culture; this is sufficient to allow for growth under OA (Melzner et al. 2011; Thomsen et al. 2013). Each experimental tank contained 30 mussels (eight 6‐L tanks per treatment, ~240 mussels in total at the start); this was the appropriate (maximum) number of mussels for each 6‐L experimental tank to maintain sufficient dissolved oxygen concentration (tested prior to experiment). For each treatment, four individual mussels were sampled from 4 separate 6‐L tanks supplied by water across the two sump systems or header tanks, required to maintain long‐term experiments (Cornwall and Hurd 2015).

### Experimental culture

Seawater *p*CO_2_ concentrations were brought to experimental levels (380, 550, 750, and 1000 *μ*atm *p*CO_2_) over a 1‐month acclimation period. Two sump systems or header tanks fed 8 separate tanks per treatment, via addition of CO_2_ mixed into air lines supplying all experimental tanks, and monitored using gas analyzers (Findlay et al. 2008; Fitzer et al. 2014b). Seawater sump systems were topped up with a mixture of seawater and fresh water once a week to simulate freshwater pulses experienced by mussels in their natural environment. This is reflected in calcite (Ω Ca) and aragonite (Ω Ar) saturation states, which are similar to other ocean acidification studies examining brackish water environments (Thomsen and Melzner 2010) and the natural variability present at the collection site (Fitzer et al. 2014a,b, 2015). Seawater salinity, temperature, and dissolved oxygen (DO) were checked daily and recorded once a week (YSI Pro2030). Seawater samples were collected (once per month) for subsequent total alkalinity (A_T_) analysis via semi‐automated titration (Metrohm 848 Titrino plus) (Dickson et al. 2007) combined with spectrometric analysis using bromocresol indicator (Yao and Byrne 1998) following methods of Fitzer et al. (2014a) and dissolved inorganic carbon (DIC) using an Automated Infra Red Inorganic Carbon Analyzer (AIRICA, Marianda instruments). Certified seawater reference materials for oceanic CO_2_ (Batch 137, Scripps Institution of Oceanography, University of California, San Diego) were used as standards to quantify the error of analysis (measured TA *μ*mol kg^−1^, DIC *μ*mol kg^−1^, CRM values TA, and DIC *μ*mol kg^−1^) (Dickson et al. 2007). Seawater A_T_, DIC, salinity, temperature, and *p*CO_2_ were used to calculate other seawater parameters using CO_2_SYS (Riebesell et al. 2007) (Table 1).

**Table 1:** Experimental seawater chemistry parameters: salinity, dissolved oxygen (DO), *p*CO_2_, total alkalinity, DIC (A_T_ and DIC ± standard deviation, SD, from the mean). Loch Fyne natural seawater chemistry parameters. Salinity, DO, and temperature are averages collected manually throughout experiments, and *p*CO_2_ given is the averaged values logged throughout the 6 months of experiments (logging every five minutes) using LI‐COR ^®^ software. Bicarbonate (${\text{HCO}}_{3}^{-}$) and carbonate (${\text{CO}}_{3}^{2-}$), calcite saturation state (Ω Ca), and aragonite saturation state (Ω Ar) were calculated from measured parameters using CO_2_Sys

Experimental condition	Salinity (ppt)	DO (%)	pHt	Temperature (°C)	*p*CO_2_ (*μ*atm)	DIC (*μ*mol kg^−1^)	A_T_ (*μ*mol kg^−1^)	${\text{HCO}}_{3}^{-}$ (*μ*mol kg^−1^)	${\text{CO}}_{3}^{2-}$ (*μ*mol kg^−1^)	Ω Ca	Ω Ar
380 *μ*atm ambient	33.23 ± 0.90	97.20 ± 0.36	6.46	11.47 ± 0.47	359.73 ± 3.54	1059.00 ± 16.83	802.54 ± 67.82	797.93	1.47	0.04	0.02
550 *μ*atm ambient	36.63 ± 1.28	98.80 ± 0.67	6.76	12.05 ± 0.36	504.47 ± 45.72	951.45 ± 2.05	830.52 ± 70.18	821.51	2.92	0.07	0.05
750 *μ*atm ambient	31.55 ± 3.50	99.38 ± 0.88	7.31	12.27 ± 0.45	732.67 ± 9.89	1227.20 ± 2.26	1202.74 ± 101.63	1159.33	15.08	0.37	0.23
750 *μ*atm ambient plus 2°C	41.80 ± 3.25	97.02 ± 0.74	6.76	14.63 ± 0.19	732.67 ± 9.89	1084.40 ± 14.05	960.16 ± 81.13	944.82	4.73	0.11	0.07
1000 *μ*atm ambient	36.96 ± 4.70	98.78 ± 0.58	6.70	12.17 ± 0.47	1131.1 ± 97.48	1325.10 ± 4.24	1136.14 ± 96.00	1123.91	4.11	0.10	0.06
1000 *μ*atm ambient plus 2°C	43.45 ± 2.83	96.83 ± 1.02	7.33	14.10 ± 0.41	1131.1 ± 97.48	1330.25 ± 2.62	1328.063 ± 112.22	1258.04	23.70	0.53	0.34
Loch Fyne Variability	19.33 ± 7.46	99.36 ± 12.99	7.93	15.70 ± 4.15	341.17 ± 102.57	1198.1	1261.95 ± 416.39	1170.56 ± 430.42	34.37 ± 18.99	0.88 ± 0.47	0.52 ± 0.29
Loch Fyne (Lowest total alkalinity values)	17.80	116	7.91	12.80	250.8 ± 3.7	836.8	876.10 ± 12.62	798.39 ± 11.75	29.19 ± 0.44	0.68 ± 0.01	0.39 ± 0.01

### Shell preparation for growth analyses

Mussels sampled after 9 months of experimental culture were dissected and shells cleaned and oven‐dried by incubation at 60°C for 48 h and then embedded in epoxy resin (EpoxyCure, Buehler) blocks. Embedded shells were sliced transversely using a diamond trim saw blade to section the whole length of the shell. New growth was determined through calcein staining of growth bands at the start of experimental culture as detailed by Fitzer et al. (2014b); any growth prior to this stained growth band is termed old growth which occurred prior to experimental culture. Resin blocks were ultra‐polished using aluminum oxide (0.3 and 1 *μ*m) and colloidal silica (0.6 *μ*m). Shell aragonite and calcite thickness were determined using light microscopy, and aragonite/calcite ratios were calculated for comparison between populations of mussels (*n* = 4) in each experimental condition. General linear model (GLM) ANOVAs (Minitab 17.1.0) were used to assess the significance of the effect of *p*CO_2_ and increased temperature on aragonite and calcite thickness and aragonite/calcite ratio with assumptions of normality and homogeneity of variance being met. Graphical methods (residual plots) were used to confirm that the data fitted a normal distribution using a frequency residual histogram and normal probability plot in Mintab 17.1.0 for the GLM ANOVA.

### Shell thickness index

Dried mussel shells were used to determine shell thickness index (STI) using the methods of Freeman and Byers (2006). STI was determined for dried shells (*n* = 4) for each treatment using the equation below:

$$\text{STI}=1000^{*}\text{dry shell weight}/\left[L^{*}{\left(H^{2}+W^{2}\right)}^{0.5}*\pi /2\right]$$

*L* = length, *H* = height, and *W* = width of dried mussel shell. The measurements were taken using a digital electronic caliper and the dry shell weight measured using a Mettler AT100 mass balance. STI is considered a good measure of shell thickness in the blue mussel with lower measurement error compared to direct measurement (Freeman and Byers 2006; Naddafi and Rudstam 2014). A high STI indicates a thicker shell, and a low STI indicates a thinner shell. STI was analyzed using a one‐way ANOVA to compare STI values between treatments and temperatures.

### Shell shape analyses

**Figure 1:**
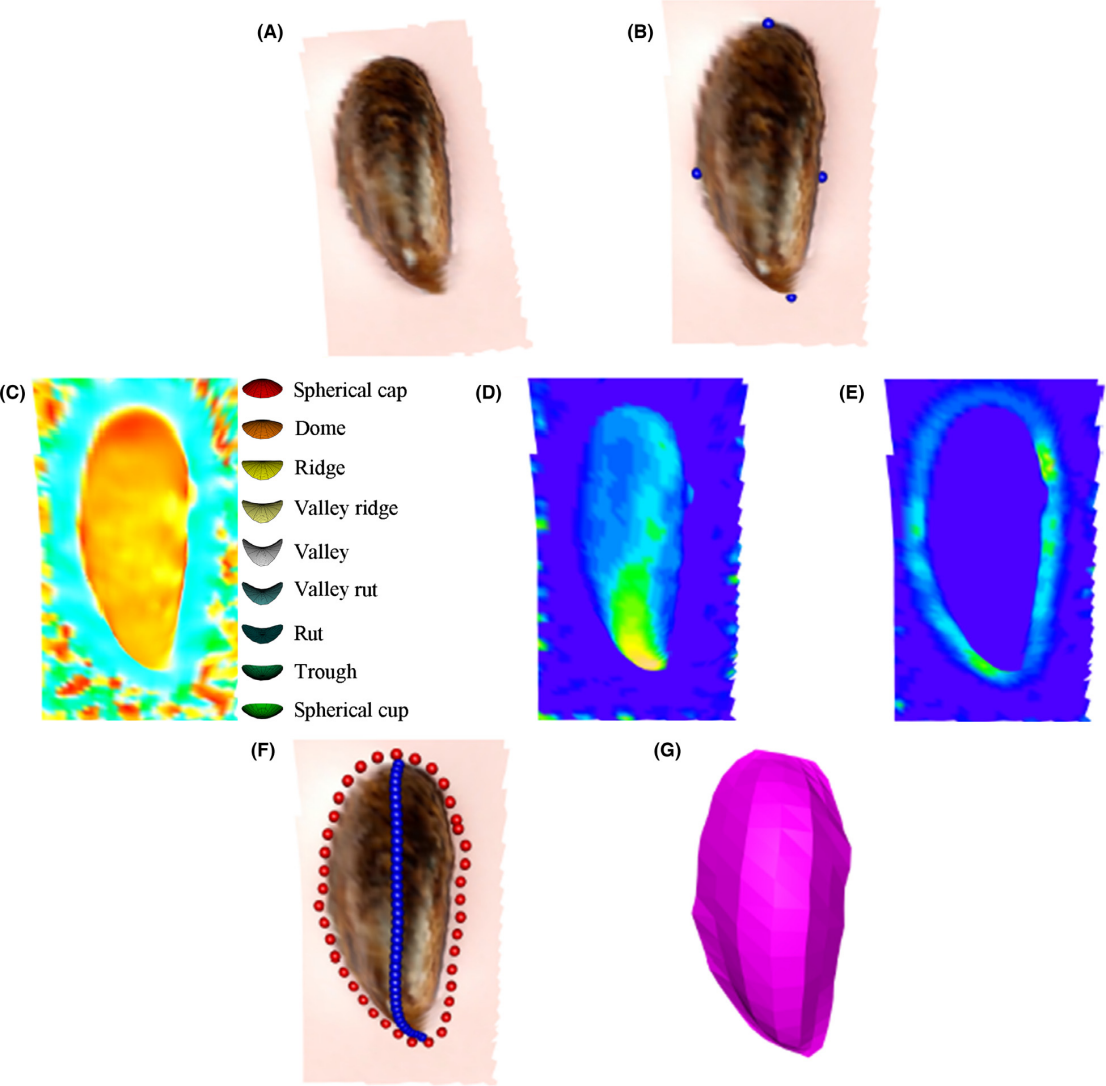
Shape analysis allocation of mussel shell ridge, perimeter, and surface representation shown for, (A) 3D image of mussel shell, (B) manual landmarks, (C) shape index of mussel shell (colored according to the color key from green (cup) through blue (valley) and yellow (ridge) to red (cap)), (D) ridge curvature values (colored topographically), (E) perimeter curvature values (colored topographically), (F) ridge and perimeter curves, and (G) rendered surface representation of mussel shell (*n* = 4 per treatment).

Mussel shells used for STI analyses were also used for shape analyses for each experimental condition (*n* = 4). One valve of the bivalve shell was attached to an imaging board with a pastel colored background. Each mussel shell was photographed using a stereo camera to produce a 3D image (Fig. 1A). Statistical shape analysis was used to describe the differences in mussel shell shape grown in the range of CO_2_ concentrations. Manual landmarks were assigned where the ridge meets the perimeter of the shell and approximately halfway in between those two locations (Fig. 1B). In order to compare the full mussel shapes, a surface representation in 3D, using the ridge and perimeter of the mussel shape (Fig. 1G), was found. Shape index is a quantifiable measure of the type of curvature at any point (concave or convex) on a surface (Fig. 1C). The principle curvatures (*k*_1_, *k*_2_), independent of location and orientation, characterize the nature and strength of the curvature at any point on the surface. Specifically, shape at any point of the surface can be described as:

$$z=\frac{1}{2}\left(k_{1}x^{2}+k_{2}y^{2}\right)$$

where the orthogonal axis *x* and *y* lie on the tangent plane (width and length of shell) and the *z* axis lies on the normal plane (depth of shell) (Fig. 1, Supporting information). The tangent plane axes correspond to the directions of the principle curvatures (*k*_1_ and *k*_2_), also called the rates of bending (Huang et al. 2006). Shape index and curvature values were calculated for each mussel (Fig. 1C,D and E). The ridge and perimeter of the mussel shell were then found by identifying the curves that pass through the locations with greatest curvature (negative and positive) of the shell surface (Fig. 1D and E). A full surface representation (Fig. 1G) was then found by triangulating from intermediary curves between the ridge and perimeter curves. This process was replicated for each mussel shell (*n* = 4) grown under each experimental condition.

**Figure 2:**
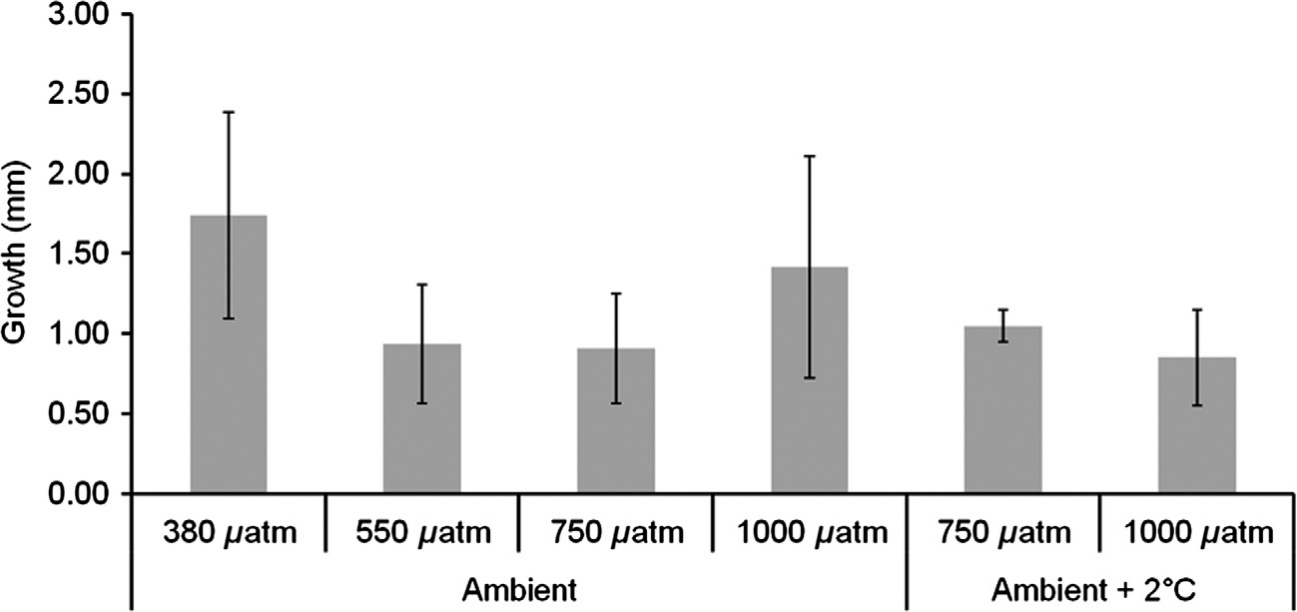
Mussel shell growth (mean ± SD) during 9 months of experimental culture (*n* = 4 per treatment). Shells were grown at 380, 550, 750, and 1000 *μ*atm CO_2_ at ambient temperature and 750 and 1000 *μ*atm CO_2_ ambient temperature + 2°C.

Generalized Procrustes analysis was applied in the statistical computing environment R (Version 3.1.1) (R Core Team, 2013), with a scaling adjustment for size in order to compare only the shape differences between the mussel shells. Visually, there is no discernible difference between the curves, either ridges or perimeters (Fig. 2, Supporting information). Principle components analysis was used to distinguish differences for curves, ridges, and perimeters between populations of mussels grown under experimental conditions (380, 500, 750, 1000 *μ*atm *p*CO_2_, 750 and 1000 *μ*atm *p*CO_2_ + 2°C), more rigorously. A regression analysis was then performed to compare any shape change of ridges, perimeters, and rendered surfaces between each population of mussel shells grown under experimental conditions (380, 500, 750, 1000 *μ*atm *p*CO_2_, 750 and 1000 *μ*atm *p*CO_2_ + 2°C). Analysis was performed to determine any difference in splay at the edge of the ridge of the mussel shell (Table 2).

**Table 2:** Profile of *z*‐direction gradients of splay in mussel shell ridge edge

Mussel	380 *μ*atm	550 *μ*atm	750 *μ*atm	750 *μ*atm + 2°C	1000 *μ*atm	1000 *μ*atm + 2°C
1	−4.25	−4.40	−5.12	−4.76	−4.64	−3.32
2	−4.04	−4.17	−4.66	−4.86	−3.48	−4.28
3	−4.43	−4.15	−4.24	−4.67	−4.43	−3.10
4	−6.63	−4.84	−3.95	−4.64		−4.21

## Results

**Figure 3:**
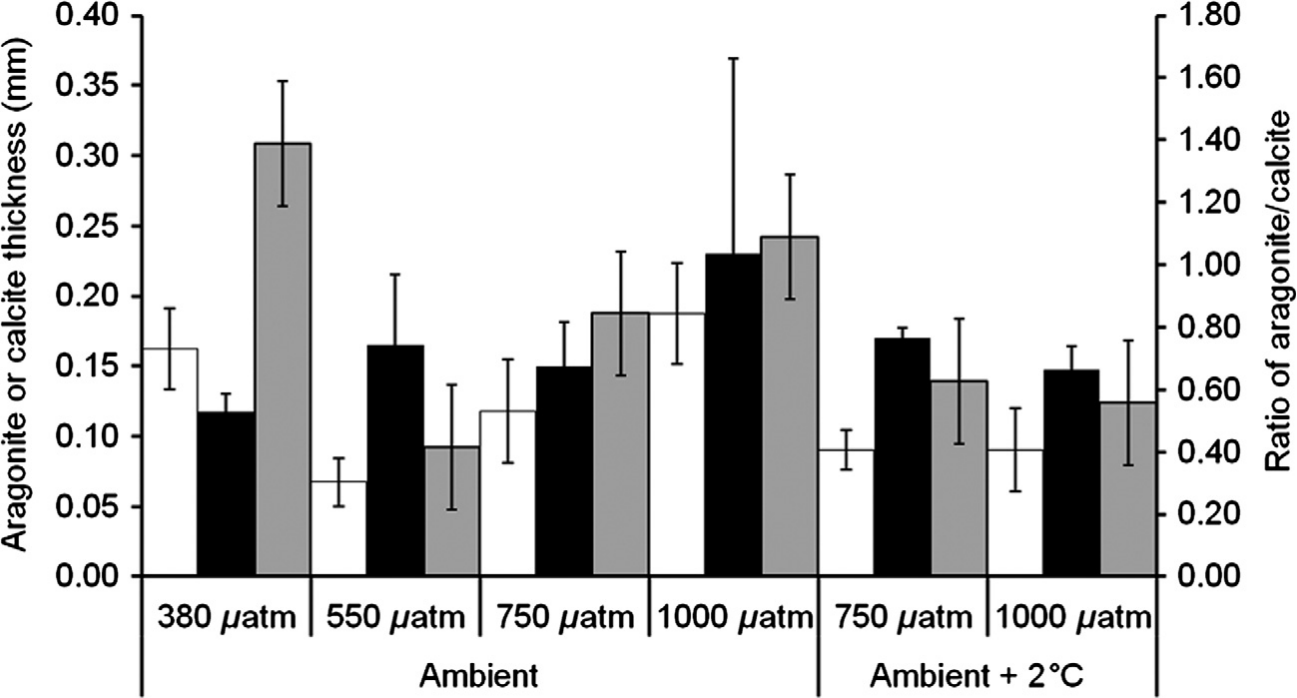
Mussel shell calcite and aragonite in new growth (mean ± SD) (*n* = 4 per treatment). Aragonite (white bars) and calcite (black bars) in millimeter and the ratio of aragonite/calcite (gray bars). Shells were grown at 380, 550, 750, and 1000 *μ*atm CO_2_ at ambient temperature and 750 and 1000 *μ*atm CO_2_ ambient temperature + 2°C.

Mussel shells cultured under ocean acidification for 9 months did not show significant reduction in growth through general linear model analysis comparing *p*CO_2_ treatments (*F* = 2.12, *P* = 0.138, df = 3, *n* = 4) with no effect of increased temperature (*F* = 0.61, *P* = 0.447, df = 1, *n* = 4) (Fig. 2). However, when examining calcite and aragonite layers of growth within the shell grown during experimental culture, ocean acidification significantly reduces the aragonite layer thickness confirmed by general linear model analysis comparing *p*CO_2_ treatments (Fig. 3) (*F* = 8.16, *P* = 0.002, df = 3, *n* = 4) with increasing temperature significantly reducing the aragonite layer further (*F* = 11.7, *P* = 0.003, df = 1, *n* = 4). The calcite layer within the new growth of the mussel shells remains similar in thickness under all experimental *p*CO_2_ concentrations confirmed by general linear model analysis comparing *p*CO_2_ treatments (*F* = 0.99, *P* = 0.423, df = 3, *n* = 4), and temperature increases (*F* = 0.02, *P* = 0.878, df = 1, *n* = 4). When combining the impact of ocean acidification and increase in temperature on the aragonite/calcite growth ratio, ocean acidification significantly reduced the aragonite/calcite ratio and so thickness of the mussel shell (*F* = 6.27, *P* = 0.005, df = 3, *n* = 4). However, temperature also significantly reduced the ratio of aragonite/calcite in the shells (*F* = 6.40, *P* = 0.022, df = 1, *n* = 4) (Fig. 3).

**Figure 4:**
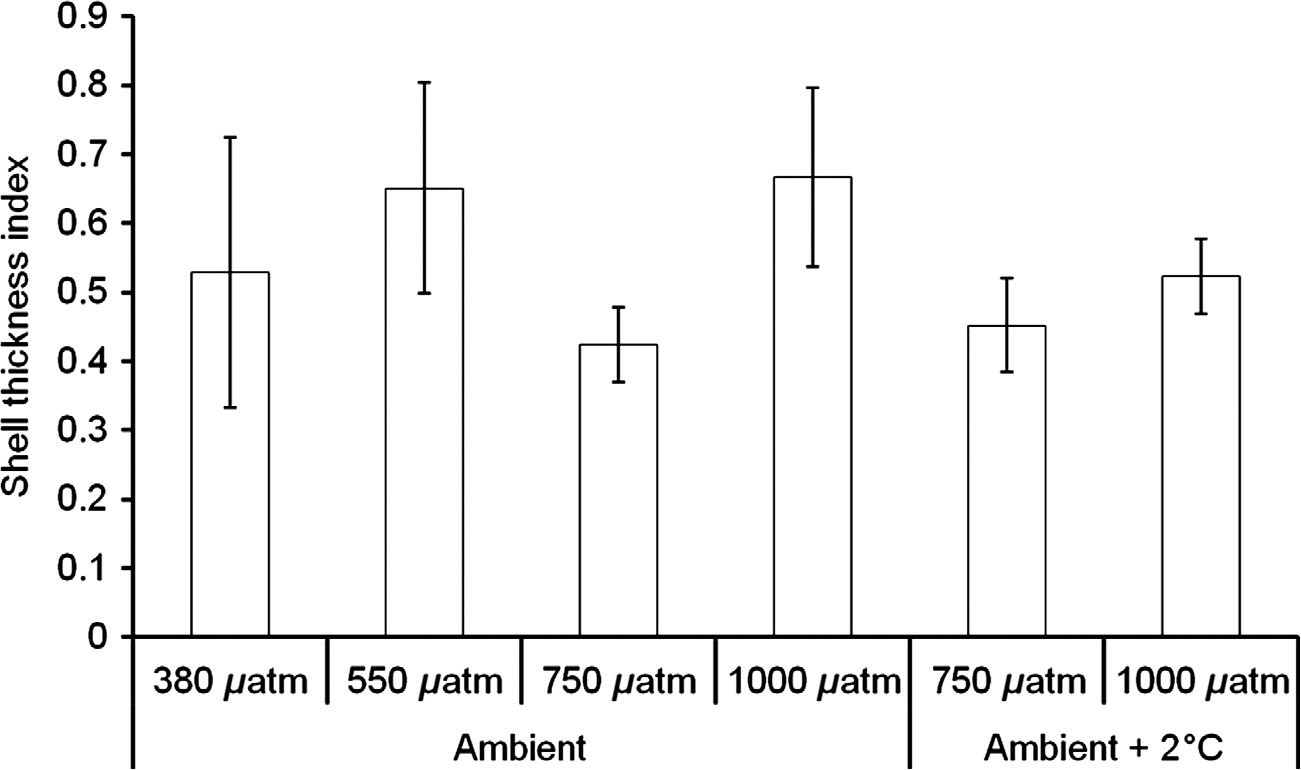
Shell thickness index (STI) for mussel shells grown under experimental conditions for 9 months (*n* = 4 per treatment). A higher shell thickness index indicates a thicker shell. Shells were grown at 380, 550, 750, and 1000 *μ*atm CO_2_ at ambient temperature and 750 and 1000 *μ*atm CO_2_ ambient temperature + 2°C.

STI, considered a good measure of shell thickness with minimized measurement error, in *M. edulis* highlights reductions in shell thickness at 750 *μ*atm *p*CO_2_ (*F* = 3.36, *P* = 0.039, df = 3, *n* = 4), whereas at 550 and 1000 *μ*atm *p*CO_2_ STI remains at similar values compared to present‐day conditions (380 *μ*atm *p*CO_2_) (Fig. 4). Temperature had no impact on STI (*F* = 1.71, *P* = 0.204, df = 1, *n* = 4) (Fig. 4).

The mussel shell ridge edge splay was compared using generalized Procrustes analysis indicating that as *p*CO_2_ increases the gradient of the *z*‐direction becomes less steep (Fig. 3A, electronic supplementary materials), although this is not statistically significant (Table 2).

**Figure 5:**
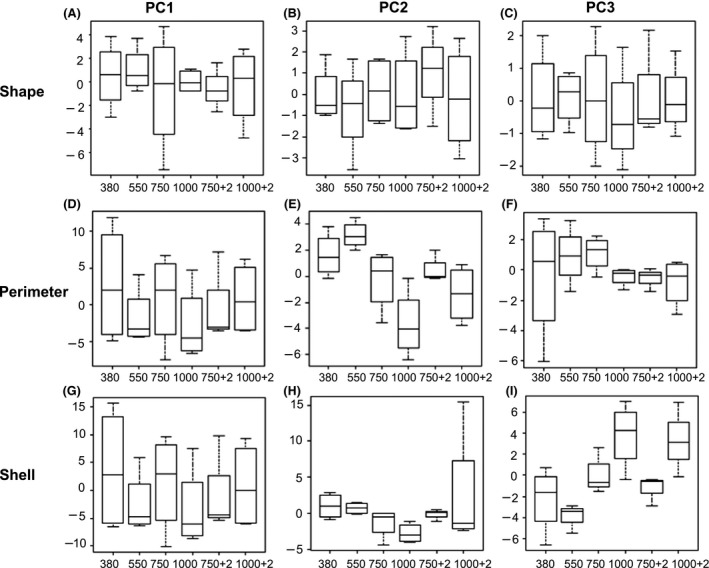
Box plots of principle components analysis of the mussel shell shape for shell ridge comparison, (A) first principal component score (*y*‐axis), (B) second principal component score (*y*‐axis), and (C) third principal component score; for shell perimeter comparison, (D) first principal component score (*y*‐axis), (E), second principal component score (*y*‐axis), and (F), third principal component score; and for shell mesh comparison for, (G) first principal component score (*y*‐axis), (E) second principal component score (*y*‐axis), and (F) third principal component score each compared across the experimental condition populations 380, 550, 750, and 1000 *μ*atm *p*CO_2_, and 750 and 1000 *μ*atm *p*CO_2_ + 2°C (*n* = 4 per treatment).

Changes to *M. edulis* shell shape were analyzed using principal components analysis which identified little difference between populations of experimental conditions with the exception of significant differences with increasing *p*CO_2_ in the third principal component of the rendered mussel shell mesh (*P* = 0.020, df = 3, *n* = 4) (Fig. 5I) and in the second component of mussel shell perimeters (*P* = 0.021, df = 3, *n* = 4) (Fig. 5E). The significant difference in the third components of the rendered mussel surfaces is due to a rise and fall of the mussel shell ridge, suggesting that the intensity of the mussel shell ridge decreases or flattens out with increasing *p*CO_2_. The second component of the mussel shell perimeter describes a distinct trend toward becoming rounded as the *p*CO_2_ increases compared to a more elliptical shape in ambient conditions (380 *μ*atm *p*CO_2_).

When comparing the regression analysis of the mussel shell shape, there was no significant overall change in mussel shell ridge (*P* = 0.835, df = 3, *n* = 4), mussel shell perimeter (*P* = 0.107, df = 3, *n* = 4), or mussel shell mesh shape (*P* = 0.288, df = 3, *n* = 4) (Fig. 4, Supporting information) between populations of experimental conditions except for the components discussed earlier for principle components analysis.

## Discussion

Shell shape analysis indicates splaying at the edge of the shells grown under ocean acidification scenarios. Although not statistically significant, this splaying could have further implications for shell protective function as splaying could result in a poorer seal between shell valves in those mussel shells grown under ocean acidification. During low tide in tidal estuarine habitats, this splaying could potentially cause problems for desiccation as a poor seal could allow air to enter the shell. There is an indication of a flattening of the shell with increasing *p*CO_2_ confirmed by the significant difference in the third component analysis of the mussel shell meshes. Differences in shell morphology due to predator cues have been reported as a defensive mechanism including production of a more rotund shell (Brönmark et al. 2011; Naddafi and Rudstam 2014). A more rotund shell, rounder and fatter in shape, reduces predator efficiency (Brönmark et al. 2011). The freshwater snail *Radix balthica* produced a more rotund shell with a low spire in the presence of fish (Brönmark et al. 2011), and the sea snail *Nucella lamellosa* developed a thicker rotund shell in the presence of crab predators (Naddafi and Rudstam 2014). In combination with a significant change in the shape of the mussel shell perimeter, becoming more circular or splayed with increasing *p*CO_2_ suggests that mussel shells grown under ocean acidification could alter their shape to potentially compensate for significant reductions in shell thickness to combat predation.

Calculated mussel shell thickness index correlates well with measured shell thickness (Freeman and Byers 2006), in this study both indicate that in ocean acidification scenarios, mussel shells are thinner. However, the measurements compared to the index also indicate reduced aragonite thicknesses at lower and higher levels of *p*CO_2_ (550 and 1000 *μ*atm *p*CO_2_, respectively). STI has been used to determine the response of the quagga mussel, *Dreissena rostriformis bugensis*, zebra mussel, *Dreissena polymorpha,* and common blue mussel *M. edulis* to invasive predators (Freeman and Byers 2006; Naddafi and Rudstam 2014). *Mytilus edulis* produced a thicker shell with increasing STI in response to predator cues (Freeman and Byers 2006), similar to *D. polymorpha,* with less thickening of shells in *D. rostriformis* (Naddafi and Rudstam 2014). The thickening of shells as a protective phenomenon is well known, and it is likely to be influenced by the evolutionary history of ecological species interactions (Freeman and Byers 2006). Changing environments as a result of climate change and, in particular, ocean acidification appears to reduce the STI of *M. edulis* shells therefore potentially reducing this protective phenomenon. Shell strength and thickness were reduced under ocean acidification in the mollusk *Austrocochlea porcata* (Coleman et al. 2014). However, in the same study, the mollusk *Subninella undulata* experienced a compensated calcification increasing the thickness and shell strength under ocean acidification (Coleman et al. 2014). In this study, *M. edulis* decreased shell thickness similar to the mollusk *A. porcata*, which could suggest a similar reduction in strength leading to impacts on survival and protection against predators (Coleman et al. 2014). Fitzer et al. (2015) observed a significant reduction in mussel shell fracture toughness and hardness (Fitzer et al. 2015). Such a change in material properties would suggest that, under ocean acidification, mussel shells become thinner and more vulnerable to damage and shell fracture.

The changes to shell morphological plasticity observed under ocean acidification in *M. edulis* here are based on experimentally simulated levels of static reduced pH. The reduced pH in correspondence to increasing *p*CO_2_ (550, 750, and 1000 *μ*atm *p*CO_2_) levels and simulated natural variability of Loch Fyne (in relation to freshwater and seawater input and seasonal temperature and light variability) remains static throughout the experiments. In the natural environment, in coastal regions of mussel cultures such as the Baltic Sea, pH is highly variable (Jansson et al. 2013). The survival and development of the bivalve *Macoma balthica* (L.) is reduced when grown under severe reductions in pH (pH 7.2, 7.4, and 7.7) fluctuating in the natural Baltic Sea environment. The low buffering capacity of the Baltic sea is expected due to its low total alkalinity (Jansson et al. 2013) which is similar to the Loch Fyne environment where the *M. edulis* specimens were originally sampled for this study. Although naturally subjected to highly variable temperatures, pH, and salinities, the additional impact of ocean acidification has severe implications for reduced survival and growth of *M. balthica* larvae (Jansson et al. 2013). Similarly, ocean acidification conditions impact shell shape and thickness of the *M. edulis* shells in this study. In the natural culture environment of Loch Fyne and the Baltic Sea, salinity is highly variable due to freshwater input (Jansson et al. 2013). Salinity was relatively static with high salinities throughout the duration of the experiment. The influence of static versus variable salinity on cadmium toxicity to the dogwhelk *Nucella lapillus* (L.) revealed that cadmium availability was significantly increased in lower fixed salinity (22 psu) or variable salinities (Leung et al. 2002). This may suggest that the salinity variability in the Loch Fyne environment may amplify the effects observed on shell shape phenotypic plasticity in static high salinity and ocean acidification alone. The mussel *Perna viridis* doubled algal feed clearance rates at 25–35 psu compared to the oyster *Crassostrea virginica*, opening its valve in fluctuating salinity conditions (5–35 psu) and achieving hemolymph osmolality equilibrium (McFarland et al. 2013). Under lower and variable salinities, the mussel valves remain closed and normal physiological processes are inhibited (McFarland et al. 2013); the oysters subjected to salinities of 0–40 psu would remain at hemolymph osmolality at much lower salinites. Mussels like *P. viridis* inhabit coastal environments often at the mouths of estuaries where the salinity is much higher than 35 psu (McFarland et al. 2013) and are less able to cope with variable salinities below the range 25–35 psu (McFarland et al. 2013). In this study, the fixed salinities were higher than the 35 psu, and so it could be suggested that the shell shape plasticity observed under ocean acidification might be amplified in combination with lower fluctuations in salinity in the natural Loch Fyne environment.

The data presented here indicate that, in addition to producing thin shells, ocean acidification induces formation of a rounder shell shape. Ocean acidification‐induced changes in shell shape indicate that rounder flatter shells could be produced by mussels as a compensation mechanism to enhance protection against predators (Brönmark et al. 2011; Naddafi and Rudstam 2014) and changing environments when mussels are unable to growth thicker shells. Changes to shell morphology can vary between species of mollusk which could impact predator–prey relationships as some mollusk species are less resistant to ocean acidification than others (Coleman et al. 2014). The use of shell shape analysis presented here is a means of determining the impact of ocean acidification on the ability of shells to defend against predation. The shell shape analysis techniques could be applied to other shell‐producing organisms to determine future ocean acidification impact on predation, which is a key process controlling population dynamics.

## Conflict of Interest

None declared.

## Data accessibility

Data available from the Dryad Digital Repository: http://dx.doi.org/10.5061/dryad.74ms0.

## Supporting information


**Figure S1.** The orthogonal axis *x*,* y* lie on the tangent plane and the *z* axis lies on the normal plane.
**Figure S2.** A diagrammatic output for the Generalised Procrustes analysis for (A) mean of mussel shell ridges, (B) mean of mussel shell perimeters, (C) extremes of mussel shell meshes, and (D) extremes of mussel shell perimeters.
**Figure S3.** A diagrammatic output for the Generalised Procrustes splay analysis for (A) mean of mussel shell ridge edge splay in 3D, (B) regression analysis output of splay in the *x*,* y*, and *z* directions, (C) 2D smoother ridge curvature for all mussels analysed, and (D).
**Figure S4.** Regression analysis output of pCO_2_ concentration against mussel shell shape changes for (A) mussel shell ridges, (B) mussel shell perimeters, and (C) mussel shell meshes.
