# Supplementary material for: Ocean acidification and temperature increase impact mussel shell shape and thickness: problematic for protection?
Source: Ecol Evol. 2015 Oct 12;5(21):4875–84. doi: 10.1002/ece3.1756 (PMC4662322; doi:10.1002/ece3.1756)
Supplement: Supplementary file 1 — Figure S1. The orthogonal axis x, y lie on the tangent plane and the z axis lies on the normal plane. Figure S2. A diagrammatic output for the Generalised Procrustes analysis for (A) mean of mussel shell ridges, (B) mean of mussel shell perimeters, (C) extremes of mussel shell meshes, and (D) extremes of mussel shell perimeters. Figure S3. A diagrammatic output for the Generalised Procrustes splay analysis for (A) mean of mussel shell ridge edge splay in 3D, (B) regression analysis output of splay in the x, y, and z directions, (C) 2D smoother ridge curvature for all mussels analysed, and (D). Figure S4. Regression analysis output of pCO2 concentration against mussel shell shape changes for (A) mussel shell ridges, (B) mussel shell perimeters, and (C) mussel shell meshes. [file ECE3-5-4875-s001.docx]

**Ocean acidification impacts mussel shell shape and thickness: problematic for shell function?**

Susan. C. Fitzer^1^*, Liberty Vittert^2^, Adrian Bowman^2^, Nicholas A. Kamenos^1^, Vernon. R. Phoenix^1^ and Maggie Cusack^1^.

***Mussel shape analysis supplementary figures***


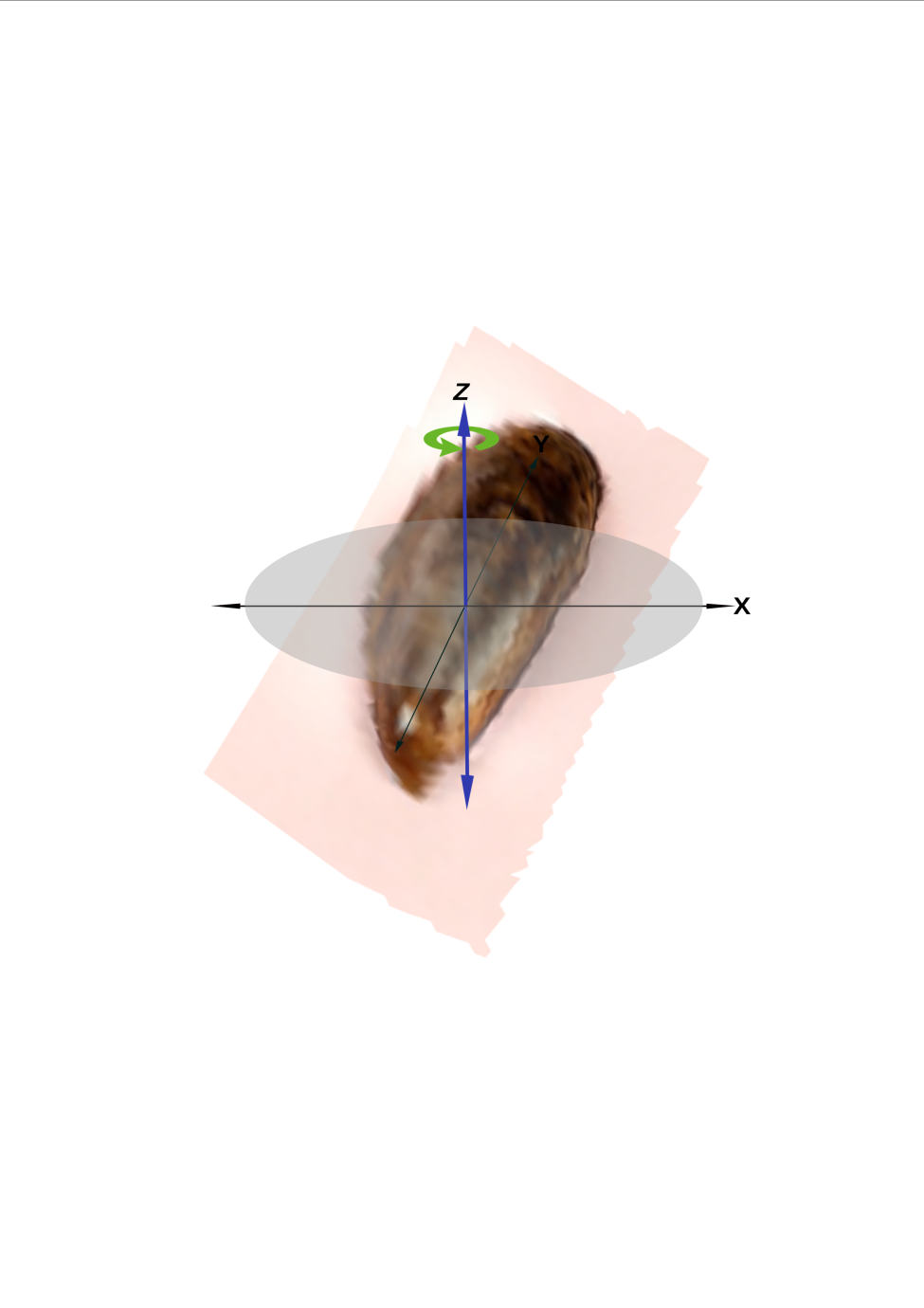


Figure 1. The orthogonal axis x, y lie on the tangent plane and the z axis lies on the normal plane. For the mussel shell shape anlaysis the orthogonal axes on the tangent plane cross the width (x) and length (y) of the mussel shell. The orthogonal axis on the normal plane crosses from the inside of the shell valve to the outside of the shell or the depth of the mussel shell (z).


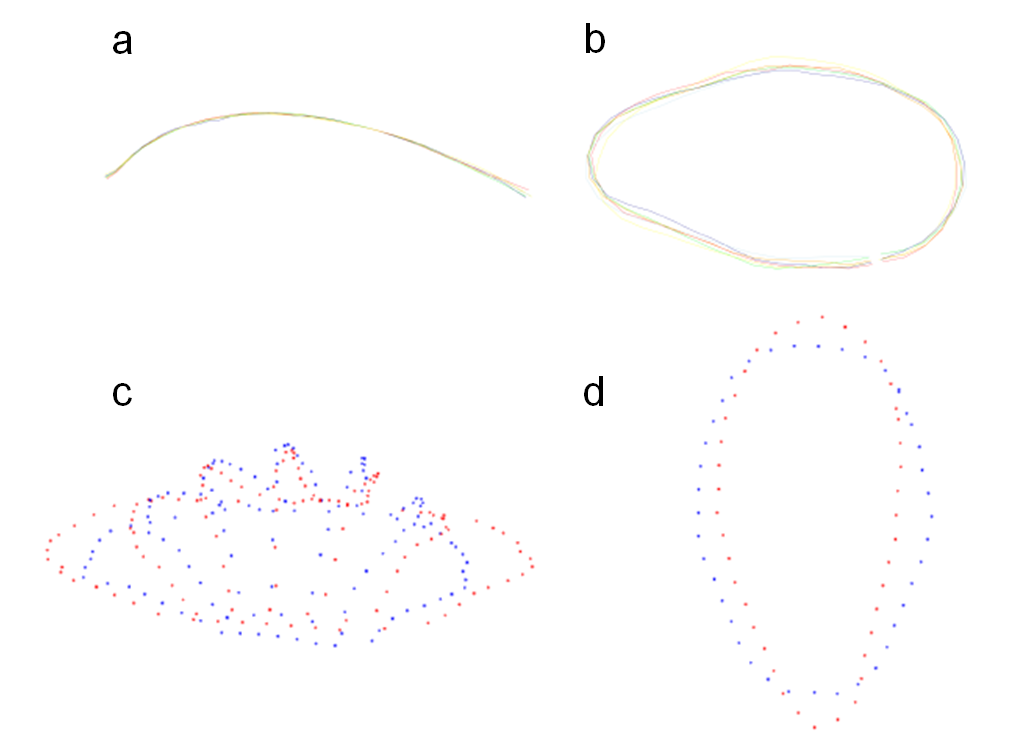


Figure 2. A diagrammatic output for the Generalised Procrustes analysis for a. mean of mussel shell ridges, b. mean of mussel shell perimeters, c. extremes of mussel shell meshes, and d. extremes of mussel shell perimeters. Note that the curves are coloured by pCO_2_ concentration for a. and b. across the experimental condition populations 380 – dark blue, 550 – light blue, 750 – green and 1000 – orange μatm pCO_2_, and 750 – yellow and 1000 – red μatm pCO_2_ + 2°C.


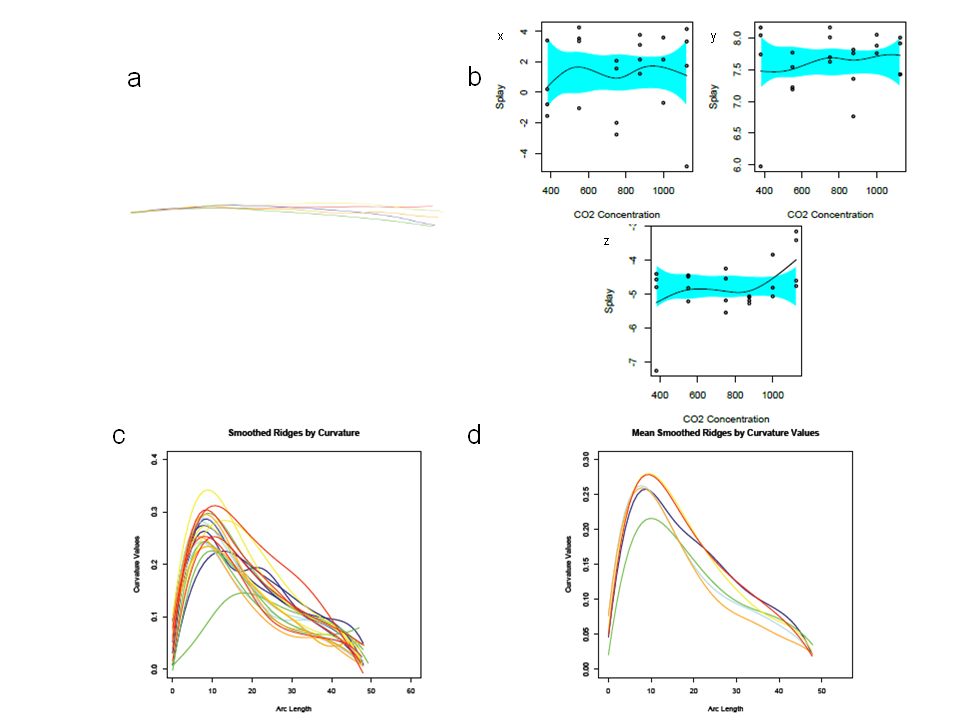


Figure 3. A diagrammatic output for the Generalised Procrustes splay analysis for a. mean of mussel shell ridge edge splay in 3D, b. regression analysis output of splay in the x, y, and z directions, c. 2D smoother ridge curvature for all mussels analysed, and d. 2D mean smoothed ridge by curvature values for each experimental condition. Note that the curves are coloured topographically by pCO2 concentration for a., c. and d. across the experimental condition populations 380 – dark blue, 550 – light blue, 750 – green and 1000 – orange μatm pCO2, and 750 – yellow and 1000 – red μatm pCO2 + 2°C.


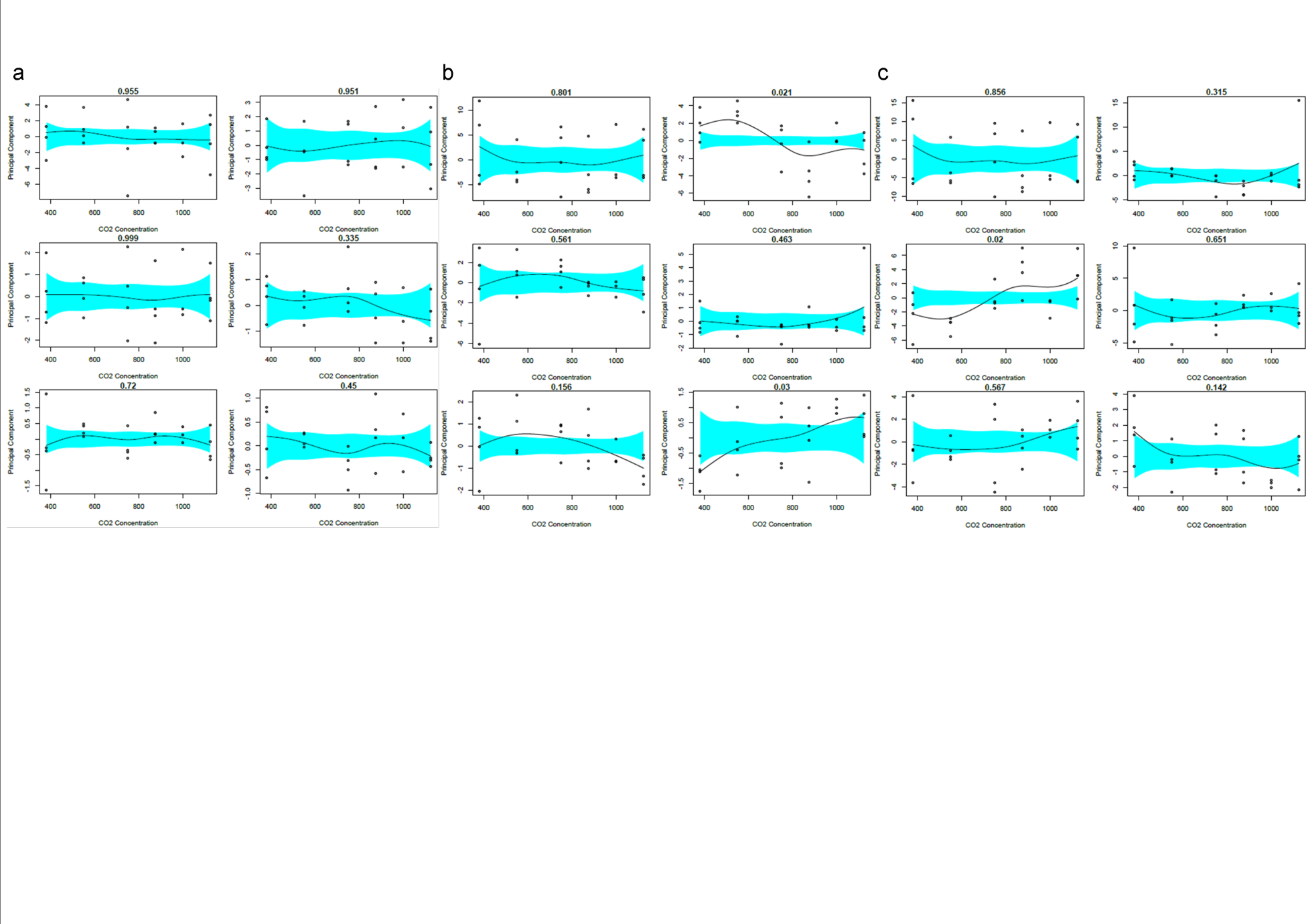


Figure 4. Regression analysis output of pCO_2_ concentration against mussel shell shape changes for a. mussel shell ridges, b. mussel shell perimeters, and c. mussel shell meshes
